# Detection of Ochratoxin a Using Molecular Beacons and Real-Time PCR Thermal Cycler

**DOI:** 10.3390/toxins7030812

**Published:** 2015-03-09

**Authors:** Simona Marianna Sanzani, Massimo Reverberi, Corrado Fanelli, Antonio Ippolito

**Affiliations:** 1 Department of Soil, Plant, and Food Sciences, University of Bari Aldo Moro, Via G. Amendola 165/A, 70126 Bari, Italy; E-Mail: antonio.ippolito@uniba.it; 2 Department of Environmental Biology, Sapienza University of Rome, P.le A. Moro 5, 00185 Rome, Italy; E-Mails: massimo.reverberi@uniroma1.it (M.R.); corrado.fanelli@uniroma1.it (C.F.)

**Keywords:** OTA detection, fluorescent dyes, *aptabeacon*, Real-time PCR thermal cycler, food safety

## Abstract

We developed a simple and cheap assay for quantitatively detecting ochratoxin A (OTA) in wine. A DNA aptamer available in literature was used as recognition probe in its molecular beacon form, *i.e.*, with a fluorescence-quenching pair at the stem ends. Our *aptabeacon* could adopt a conformation allowing OTA binding, causing a fluorescence rise due to the increased distance between fluorophore and quencher. We used real-time PCR equipment for capturing the signal. With this assay, under optimized conditions, the entire process can be completed within 1 h. In addition, the proposed system exhibited a good selectivity for OTA against other mycotoxins (ochratoxin B and aflatoxin M1) and limited interference from aflatoxin B1 and patulin. A wide linear detection range (0.2–2000 µM) was achieved, with LOD = 13 nM, *r* = 0.9952, and *R*^2^ = 0.9904. The *aptabeacon* was also applied to detect OTA in red wine spiked with the same dilution series. A linear correlation with a LOD = 19 nM, *r* = 0.9843, and *R*^2^ = 0.9708 was observed, with recoveries in the range 63%–105%. Intra- and inter-day assays confirmed its reproducibility. The proposed biosensor, although still being finalized, might significantly facilitate the quantitative detection of OTA in wine samples, thus improving their quality control from a food safety perspective.

## 1. Introduction

The food safety issue is gaining *momentum* among producers and consumers, thus, the setup of tools for the sensitive and rapid detection and quantification of specific molecules with a significance for human and environmental health in biological, environmental, and food samples is tightly required. Indeed, the traditional analytical techniques as thin-layer chromatography (TLC), high-performance liquid chromatography (HPLC) or gas chromatography (GC) coupled to mass spectrometry require expensive and time-consuming sample preparation (extraction, clean up, preconcentration and derivatization), sophisticated equipment and trained personnel [1].

Although biosensors have emerged as alternative analytical tools because of their portability, ease of use, and high sensitivity and selectivity, their applications were less numerous than expected. In fact, the use of biological recognition element (e.g., enzymes, cells, or antibodies) provided limited success because of cross reactivity and matrix interference, with consequently frequent false negative or positive results. Moreover, they strongly depend on the quality of the antibodies, and their preparation via animal immunization may cause instability or modifications [2].

Among the possible alternatives, aptamers have recently gained considerable attention. They are functional nucleic acids (DNA or RNA) obtained from combinatorial oligonucleotide libraries through *in vitro* selection, to bind to specific targets as drugs, proteins, peptides, vitamins and other organic and inorganic compounds [3]. Aptamers can successfully compete with antibodies due to their specific properties. In fact once selected, they can be synthetized at any time, in large amounts, and with reproducibility. They can bind even to not immunogenic targets, with no need for animal or cell cultures, and offer a greater surface density of receptors [2]. Finally, they are thermally stable, reusable and easily modifiable for detection and immobilization by introducing reporter molecules and functional groups.

Among the most interesting targets for an aptamer-based detection, there are the mycotoxins, secondary metabolites produced by certain fungal genera, which are toxic to humans, animals and plants, so that a role in plant diseases cannot be excluded [4]. One of the first selected aptamers targeted ochratoxin A (OTA) [5], a mycotoxin produced by various *Aspergillus* and *Penicillium* species and detected in several food matrices. In Mediterranean countries, it is mainly associated to grapes and derived products especially wine. The International Agency for Research on Cancer (IARC) classified this toxin as a potential carcinogen for humans (group 2B), so that the European Commission set up regulatory limits for raw cereal grains (5 µg/kg), dried fruits (10 µg/kg), roasted coffee (5 µg/kg), grape juice and all types of wines (2 µg/kg) [6].

Since then, many aptamer-based biosensors for OTA detection were proposed. For instance, Cruz-Aguado and Penner [7] applied the anti-OTA aptamer coupled with fluorescence polarization assays. However, the method required multiple, complex and time-consuming steps prior to detection. In 2010, Kuang *et al.* [8] proposed a “signal-off” electrochemical aptasensor with gold nanoparticles, which albeit ultrasensitive, required complex modifications of the nanoparticles and might suffer from interference from other electro-active species coexisting in real samples. Recently, Chen *et al.* [1] developed a fluorescent sensing platform based on a target-induced structure-switching signalling aptamer. Nevertheless, the sensitivity of the method was not high enough for OTA detection in real samples. Therefore, despite efforts, as far as we know, a simple, cheap and fast aptamer-based OTA biosensor, with satisfactory sensitivity and selectivity is still missing.

In our study, we combined aptamer chemistry and Real time PCR detection system to provide an assay that may result more easily affordable than HPLC equipment, especially in laboratories equipped for molecular biology. In particular, we used the OTA aptamer in a molecular beacon structure. Indeed, molecular beacons can be designed to directly detect and quantify different nucleic acid targets [9]. In absence of the target, beacons form a stem-loop structure with a fluorophore on one end of the stem and a quencher on the other end, so that the fluorophore is quenched by energy transfer. The loop contains a sequence complementary to the target, therefore in its presence, a duplex is formed and the stem is broken, separating the fluorophore from the quencher and restoring the fluorescence. We expect that, because of the higher affinity of the aptamer-loop to OTA molecules compared to that of the complementary arms, the *aptabeacon* may open emitting a fluorescence captured by the Real-time PCR equipment as in a normal amplification reaction.

## 2. Results and Discussion

**Figure 1:**
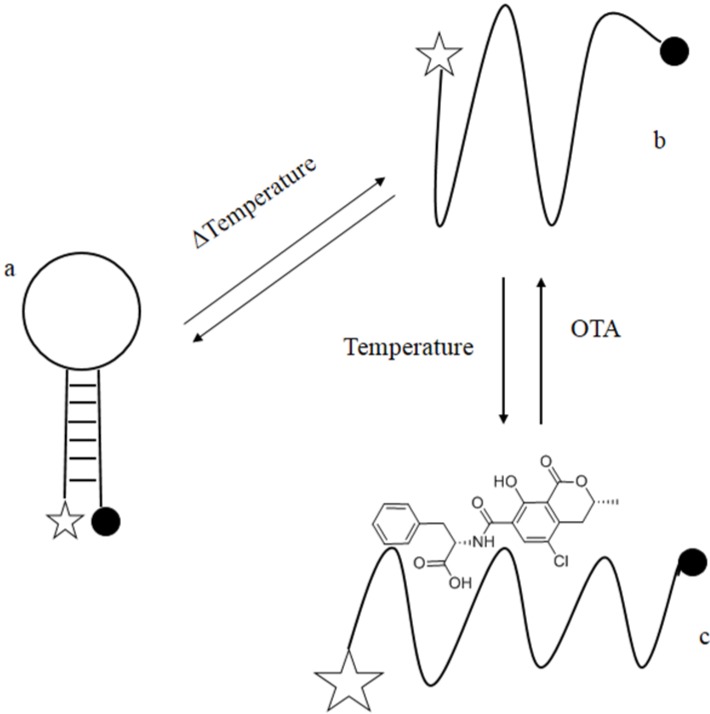
Schematic drawing of mechanism of signalling by ochratoxin A (OTA) *aptabeacon*. (**a**) *Aptabeacon* in quenched stem-loop conformation in absence of OTA; (**b**) *Aptabeacon* in unfolded conformation that allows OTA binding; (**c**) OTA bound to *aptabeacon*. Stars represent the fluorophore and the black spots the quencher. The size of the stars represents the emission intensity of the fluorophore; it increases with the distance between the fluorophore and the quencher.

The hypothesis underlying the present investigation was that additional sequences could be attached to the OTA aptamer selected by Cruz-Aguado and Penner [5] to form a stable stem-loop and destabilize the native binding structure. Similarly to molecular beacons, in absence of the target molecule, the *aptabeacon* would have maintained a quenched stem-loop structure. On the contrary, in presence of OTA the equilibrium between quenched and unquenched structures would have been shifted towards the OTA-binding conformer, generating a change in observed fluorescence intensity because of the fluorescence-quenching pair added to the 5'- and 3'-ends of the engineered stem-loop (Figure 1). Therefore, we designed 7-nucloetide long complementary arms with an 86% GC to flank the aptamer sequence in order to obtain a hairpin structure, and a 6FAM/BHQ1 as fluorophore/quencher system.

Several binding buffers, temperatures, and incubation conditions were tested in order to achieve maximum fluorescence intensity. The binding buffer selected was that reported by Chen *et al.* (2012) [1]: 10 mM Tris pH 8.5, 120 mM NaCl, 5 mM KCl, 20 mM CaCl_2_. The incubation foresaw an initial denaturation (90 °C for 5 min) followed 60 min at 30 °C; these conditions were selected on the bases of experimental evidence (data not shown) and taking into account previous similar reports, stating that at room temperature the fluorescence signal was steady up to 1 h after the first measurement. A K_d_ of 0.8 µM was calculated, which is higher than the one reported by Cruz-Aguado and Penner [5], but nonetheless satisfactory.

**Figure 2:**
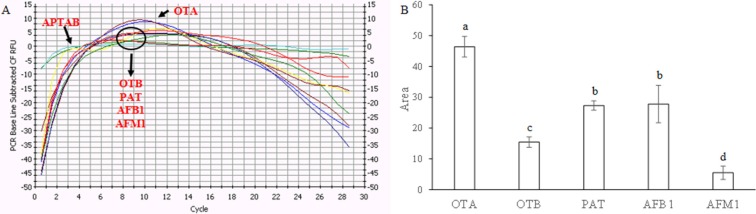
Selectivity of the aptameric assay system toward ochratoxin A (OTA) against other mycotoxins (OTB, ochratoxin B; AFB1, aflatoxin B1; AFM1, aflatoxin M1; PAT, patulin) at the same concentration (2 µM). The blank signal (*aptabeacon* in absence of OTA) was subtracted to all samples. (**A**) Instrument screen; (**B**) toxins’ peak areas. Each bar represents the average value of three independent experiments (with three technical replicates each) with error bars indicating standard error of the mean (SEM). Significant differences (*p* ≤ 0.05) were identified with the Duncan’s Multiple Range Test: bars with different letters are significantly different.

Since the selectivity is another important issue for practical implementation of OTA detectors, we compared the change of fluorescent intensity induced by other relevant mycotoxins (OTB, AFB1, AFM1, and PAT) with a potential combination ability with OTA aptamer [8]. As shown in Figure 2, at the same concentration, the aptabeacon exhibited evident different response signals to the mycotoxins. The response signals to OTB (OTA structural analogue) and AFM1 in term of peak area were on average 67–88% lower than that to OTA, whereas those of AFB1 or PAT curves were up to 41% lower. Aflatoxins have a different product range [10] and patulin, although potentially present on grapes [11] disappears rapidly during fermentation to essentially a zero level [12]. These findings, together with the difference in affinity between OTA and OTB with the aptamer, were considered even in previous similar investigations sufficient for practical application [7].

**Figure 3:**
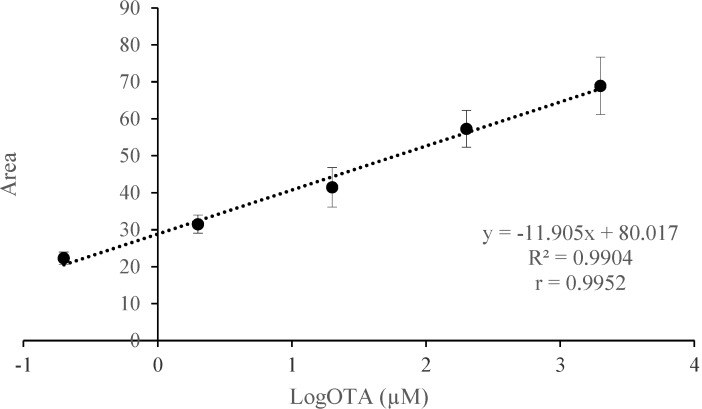
Linear relationship between OTA concentration and peak areas in the range 2000–0.2 µM. Standard curves, linear equations, and determination (*R*^2^) and correlation (*r*) coefficients were determined by plotting area values (y-axis) against log OTA concentration (x-axis). Error bars (indicating standard error of the mean, SEM) were obtained from three parallel experiments, in which each sample was run in triplicate.

The sensitivity of the *aptabeacon* was investigated by varying OTA concentration under the optimized assay conditions. As expected, the addition of an increasing amount of OTA resulted in a dynamic increase in fluorescent emission intensity at 492 nm and consequently in peak areas. In particular, a linear correlation between the peak area and the concentration of OTA was observed (Figure 3) in the range 2000–0.2 µM.

The calibration equation obtained from this curve was y = −11.905x + 80.017 with very good determination and correlation coefficients of 0.9904 and 0.9952, respectively. The calculated limit of LoD was 13 nM with a signal-to-noise ratio of 3. The recorded selectivity for OTA and LoD are satisfactorily comparable to other OTA aptamer-based detection methods reported in literature [1,13,14].

**Figure 4:**
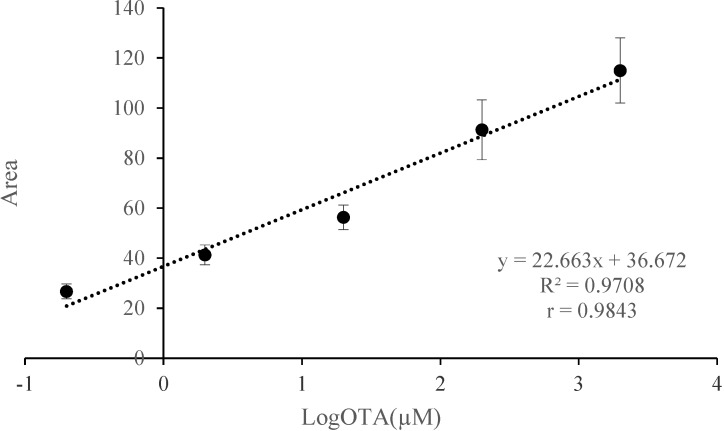
Application of aptasensor for OTA determination in spiked red wine (2000–0.2 µM, final concentration). Standard curves, linear equations, and determination (*R*^2^) and correlation (*r*) coefficients were determined by plotting area values (y-axis) against log OTA concentration (x-axis). Error bars (indicating standard error of the mean, SEM) were obtained from three parallel experiments, in which each sample was run in triplicate.

In order to evaluate the practical applicability and accuracy, the *aptabeacon* was used for determining the recoveries by spiking real red wine samples with five different OTA concentrations (4000, 400, 40, 4, 0.4 µM) so that in reaction mix the final concentrations were 2000, 200, 20, 2 and 0.2 µM. The calibration curve in shown in Figure 4.

The equation was y = 22.663x + 36.672 with satisfactory determination and correlation coefficients of 0.9708 and 0.9843, respectively. The calculated LoD was 19 nM with a signal-to-noise ratio of 3. The recovery was in the range of 63–105%, in substantial accordance with what foreseen for mycotoxin recover (70–110%) [15], and reported in similar studies [16]; it can be considered acceptable, since referred to minimally processed biological samples. The intra- and inter-day precision data are reported in Table 1. RDS calculated in intra- and inter-day assays ranged from 2.9 to 6.9, revealing a satisfying reproducibility.

Considering that, the system proved to be sensitive enough to detect OTA in wine below the regulatory limit (2 µM), although further trials are needed on real samples in a larger scale, it could be potentially applied in monitoring fungi’ toxigenic ability and consequent product contamination. To this purpose, even the lack of sample preparation represents an advantage of our assay in relation to the already published ones. Finally, since it has been reported the role of OTA in the adaptation of producing fungi to hostile environments [17], its early detection might have a significance even from a disease control perspective.

**Table 1:** Intra and interday precision for OTA measurement.

Expected Concentration (µM)	Intra-day (*n* = 5)		Inter-day (*n* = 15)	
	Measured (µM)	RSD (%)	Measured (µM)	RSD (%)
2000	2023 ± 59	2.9	2020 ± 65	3.2
200	216 ± 15	6.9	216 ± 14	6.5
20	18 ± 1	5.5	19 ± 1.2	6.3
2	1.7 ± 0.1	5.9	1.8 ± 0.1	5.5
0.2	0.18 ± 0.01	5.5	0.21 ± 0.01	4.8

## 3. Experimental Section

### 3.1. Materials and Reagents

Ochratoxin A and B (OTA and OTB, from *Aspergillus ochraceus*), aflatoxin B1 and M1 (AFB1 and AFM1, from *Aspergillus flavus*), patulin (PAT, from *Penicillium expansum*) and buffer components were purchased from Sigma-Aldrich (St. Louis, MO, USA). The resuspension solutions were 10% methanol or double-distilled water pH 4 (patulin). Stock solutions were prepared (concentration 200 mM) and then diluted to the desired concentrations.

The sequence of the OTA aptamer was that reported by Cruz-Aguado and Penner [5]. The *aptabeacon* synthesised by Sigma-Aldrich, with the OTA aptamer flanked by 7-nucleotide arms with at 5'-end the fluorophore 6-FAM and at 3'-end the quencher BHQ1, is given below:

6FAM-CGC GCT GGA TCG GGT GTG GGT GGC GTA AAG GGA GCA TCG GAC ACA GCG CG-BHQ1.

The beacon was resuspended in TE buffer pH 8.0 at concentration 100 µM, and then diluted in ultra-pure sterile water to the desired concentration.

### 3.2. Reaction Mixture and Fluorimetric Measurement

The reaction was conducted in a 20 µL volume containing: 16 µL reaction buffer, 2 µL *aptabeacon* (final concentration 1 µM) and 2 µL OTA (final concentration 2 µM). Each sample was run in three technical replicates. Different reaction buffers [1,2,13,14], temperatures (25, 30, 60, 70 °C) and times (20, 30, 60 min) of incubation or signal acquisition (30, 60, 120 s) were tested in order to achieve maximum fluorescence intensity. To assess specificity of the signal, in specific reactions ultra-pure sterile water replaced OTA or aptabeacon.

The fluorescence was captured in a 96 well-plates iCycler iQ thermal cycler (Bio-Rad, Hercules, CA, USA) by the iCycler iQTM associated software (Bio-Rad Real time Detection System Software, version 3.0). The reaction conditions were 5 min at 90 °C, followed by 30 cycles of 2 min at 30 °C.

Kinetic constant (K_d_) for OTA binding *aptabeacon* was determined using the equation: K_d_ = ([A] × [L])/[AL], where [A], [L] and [AL] represent micromolar concentrations of the aptamer, ligand and complex, respectively.

### 3.3. Specificity Analysis

To further test the specificity of the *aptabeacon*-OTA assay for highly selective detection, four other relevant mycotoxins, namely OTB, AFB1, AFM1 and PAT were used as alternative targets in the reaction at the same OTA final concentration (2 μM). The reaction mix and conditions were the same reported above.

### 3.4. Sensitivity Analysis

In order to calculate the sensitivity of the assay and establish the detection limit, OTA was added to the reaction mix to reach 2000, 200, 20, 2, and 0.2 µM as final concentrations. The reaction mixture and conditions were the same reported above.

The area underneath the curves obtained by plotting the positive relative fluorescence unit (RFU) values and the x-axis, was calculated by an integral using Graph 4.4.2 software (https://www.padowan.dk/download/). The experiment was conducted in triplicate and the average values reported ± standard error of the mean (SEM). Standard curves, linear equations and determination (*R*^2^) and correlation (*r*) coefficients were determined by plotting area values (y-axis) against log OTA concentration (x-axis).

Limit of Detection (LoD) was calculated according Armbruster and Pry [18] with the following formula:

LoD = LoB + 1.645(SD _low concentration sample_) (1)

where LoB = mean blank + 1.645(SD _blank_).

### 3.5. Analysis of Wine Samples

Non contaminated wine samples (red wine 2011, Aglianico from Castel del Monte, Italy, 12% alcohol) were passed through a 0.45 µm syringe filter (Albet, Murcia, Spain) and spiked with OTA standard to reach five concentrations (4000, 400, 40, 4, 0.4 µM). The samples were diluted 1:1 with the binding buffer so to reach five final concentrations (2000, 200, 20, 2, 0.2 µM) and added with the aptabeacon (1 µM). The reaction conditions were the same reported above. The experiment was conducted in triplicate and the average values reported ± standard error of the mean (SEM). The LoD was calculated as reported above. Recovery was calculated as follows:

R(%) = (actual analyte concentration/expected analyte concentration) × 100

Inter- and intra-day precision values using this method were estimated by assaying control wine containing five different concentrations of OTA, 5 times on the same day and on three separate days to obtain the relative standard deviation (RSD). The equation of the relative standard deviation, given as a percentage, is as follows:

(2) $$\text{RSD}(\%)=(s/\bar{x})\times 100$$

where *s* is equal to the standard deviation and $\bar{x}$ is equal to the mean.

## 4. Conclusions

According to our experimental results, although further large-scale experiments on real samples are needed, the proposed *aptabeacon* could be applied to quantitatively determine the ability of producing fungi to contaminate wine with OTA. In fact, it proved to be sensitive, simple, fast, and cheap, and as such, it might improve food products safety, especially when a large number of samples have to be examined at once.

Since the instrument needed for our assay is cheaper than an HPLC equipment and already available in many mycology laboratories and medium-sized companies and the detection is obtained because of a direct probe/analyte interaction, our method might represent a significant improvement in aptamer-based OTA detection.

Moreover, once generalized to mycotoxin detection in different matrices, molecular beacon coupled with aptamer technology might be feasibly applied to develop specific lab-on-chip devices.
